# Supplementary material for: Vitamin C supplementation reduces expression of circulating miR-451a in subjects with poorly controlled type 2 diabetes mellitus and high oxidative stress
Source: PeerJ. 2021 Feb 4;9:e10776. doi: 10.7717/peerj.10776 (PMC7868066; doi:10.7717/peerj.10776)
Supplement: Supplemental Information 5 — The data showed no significant difference of both miRNAs. [file peerj-09-10776-s005.docx]

**Supplementary data_2**


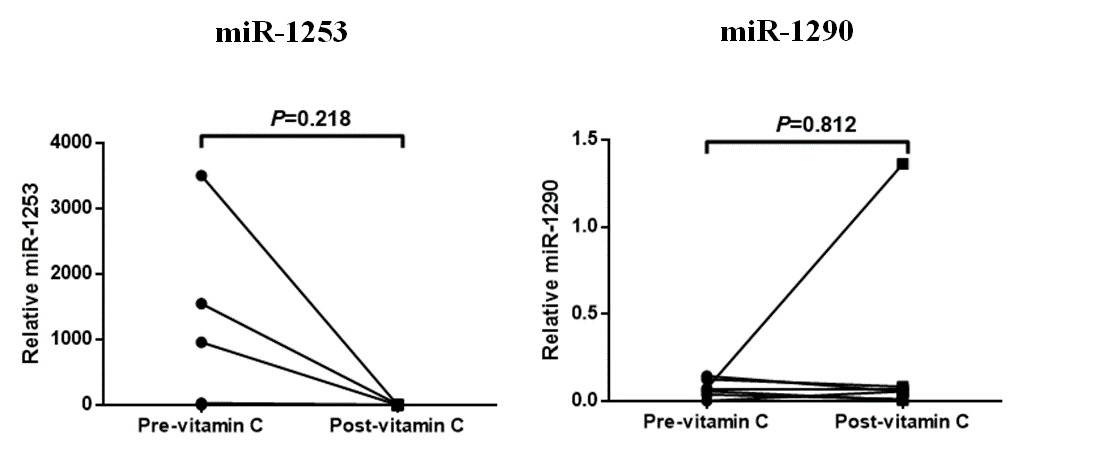


**Figure S2 qPCR data of mi-1253 and miR-1290.** The data showed no significant difference of both miRNAs.
