# Supplementary material for: Vitamin C supplementation reduces expression of circulating miR-451a in subjects with poorly controlled type 2 diabetes mellitus and high oxidative stress
Source: PeerJ. 2021 Feb 4;9:e10776. doi: 10.7717/peerj.10776 (PMC7868066; doi:10.7717/peerj.10776)
Supplement: Supplemental Information 6 — Figure S1: Number of baseline miRNAs from plasma of T2DM subjects. The numbers of baseline miRNAs are depicted on the y-axis and number of counts on the X-axis. The most miRNA counts are around 10–50. Figure S2: miR-451 expression in subjects receiving placebo control (n = 8). The plot represents relative expression of miR-451a as validated by qRT-PCR using the cel-miR-39 spike-in control as a reference gene. Data is from eight subjects from a randomized, placebo controlled, crossover design study as has been reported recently (Chuangchot et al., 2020), who have received placebo daily for six weeks and were also investigated for miR-451a expression at the pre-and post-placebo supplementation. There was no significant difference after placebo supplementation (p = 0.308). Table S1 : List of abundant plasma microRNAs at baseline of T2DM subjects Table S2 : Common validated target genes of miR-451a involved in biological processes Table S3 : Characteristics of the participants at the pre-and post-vitamin C and placebo supplementation timepoints. [file peerj-09-10776-s006.docx]

**Supplementary data**

**Figure S1 Number of baseline miRNAs from plasma of T2DM subjects.** The numbers of baseline miRNAs are depicted on the y-axis and number of counts on the X-axis. The most miRNA counts are around 10-50.


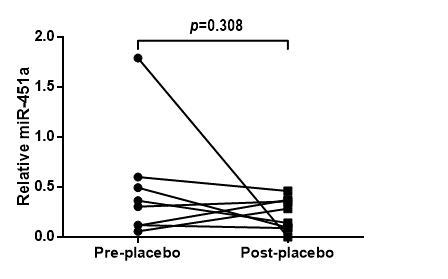


**Figure S2** **miR-451 expression in subjects receiving placebo control (n=8).** The plot represents relative expression of miR-451a as validated by qRT-PCR using the cel-miR-39 spike-in control as a reference gene. Data is from eight subjects from a randomized, placebo controlled, crossover design study as has been reported recently *(Chuangchot et al. 2020)*, who have received placebo daily for six weeks and were also investigated for miR-451a expression at the pre-and post-placebo supplementation. There was no significant difference after placebo supplementation (p=0.308).

**Table S1 List of abundant plasma microRNAs at baseline of T2DM subjects.**

| **microRNA raw counts** | **Numbers** | **microRNAs** |
| --- | --- | --- |
| >1000 | 1 | miR-451a |
| 500-1000 | 1 | miR-1253 |
| 100-500 | 24 | miR-644a, miR-302d-3p, miR-4454+ miR-7975, miR-122-5p, miR-223-3p, miR-495-3p, miR-579-3p, miR-549a, miR-548q, miR-499a-5p, miR-4536-5p, miR-1290, miR-590-5p, miR-548ah-5p, miR-548g-3p, miR-6721-5p, miR-378h, miR-3144-3p, miR-3161, miR-30e-5p, miR-2116-5p, miR-150-5p, miR-612, miR-363-3p |

**Table S2** **Common validated target genes of miR-451a involved in biological processes.**

| **Gene symbol** | **Name** | **Biological process key words** |
| --- | --- | --- |
| AKT1 | RAC-alpha serine/threonine-protein kinase | Lipid metabolism |
| ATF2 | Cyclic AMP-dependent transcription factor ATF-2 | Inflammation, Insulin resistance |
| BCL2 | BCL2 apoptosis regulator | Apoptosis, Glucose metabolism |
| BMI1 | Polycomb complex protein BMI-1 | Transcription regulation |
| BRAF | B-Raf proto-oncogene | Cell division, differentiation, and secretion |
| CAB39 | Calcium-binding protein 39 | Oxidative stress |
| GFPT1 | Glutamine--fructose-6-phosphate transaminase 1 | Hexosamine pathway |
| GNAQ | G Protein subunit alpha Q | Transmembrane signaling |
| IKBKB | Inhibitor of nuclear factor kappa-B kinase subunit beta | Inflammation, Insulin resistance |
| IL6R | Interleukin 6 receptor | Inflammation, Glucose metabolism |
| LPAR1 | Lysophosphatidic acid receptor 1 | Cell signaling |
| MMP2 | Matrix metallopeptidase 2 | Tissue remodeling, Inflammation |
| MMP9 | Matrix metalloproteinase-9 | Wound healing, Leukocyte migration |
| MYC | MYC proto-oncogene | Apoptosis, Glucose metabolism |
| PCGF5 | Polycomb group ring finger 5 | Transcription regulation |
| PIK3R1 | Phosphoinositide-3-kinase regulatory subunit 1 | Insulin signal transduction, Glucose uptake |
| PRKAA1 | Protein kinase AMP-activated catalytic subunit alpha 1 | Glucose metabolism |
| RAB14 | Ras-related protein Rab-14 | Intracellular membrane trafficking |
| STK11 | Serine threonine kinase 11 | Key regulator of the AMP-activated protein kinase (AMPK) |

**Table S3** **Characteristics of the participants at the pre-and post-vitamin C and placebo supplementation timepoints.**

| Factor | Vitamin C (n=8) | |  | Placebo (n=8) | |
| --- | --- | --- | --- | --- | --- |
|  | Pre | Post |  | Pre | Post |
| Age (year) | 58.8±5.9 | 58.8±5.9 |  | - | - |
| BMI (Kg/m^2^) | 26.2±4.1 | 26.2±3.9 |  | 25.9±3.8 | 25.3±3.7 |
| HbA1c (mmol/mol) | 95.0±15.0 | 95.0±14.8 |  | 91.7±16.8 | 96.4±15.6 |
| HbA1c (%) | 11.0±1.4 | 11.0±1.3 |  | 10.5±1.5 | 11.0±1.4 |
| FBS (mmol/L) | 13.0±4.3 | 10.0±2.0 |  | 8.6±2.2 | 8.2±1.8 |
| Plasma vitamin C levels (µmol/L) | 57.8±11.0 | 90.5±55.5* |  | 63.58±21.7 | 69.01±32.6 |
| Plasma MDA levels (µmol/mL) | 17.0±8.6 | 10.6±3.8* |  | 11.71±7.4 | 17.19±8.9^#^ |
| Plasma F_2_IsoPs (pg/mL) | 16.9±4.8 | 12.0±4.3* |  | 15.8±4.3 | 16.2±4.7 |
| Phagocytosis (%) | 20.4±8.6 | 30.7±11.4* |  | 19.0±1.9 | 19.4±8.8 |
| Oxidative burst (%) | 5.9±3.5 | 11.1±4.5* |  | 7.6±5.7 | 7.9±5.4 |
| Cholesterol (mg/dL) | 239.3±60.1 | 187.9±45.9* |  | 205.9±71.4 | 214.1±52.9 |
| Triglyceride (mg/dL) | 168.8±63.8 | 195.9±100.1 |  | 149.9±68.4 | 167.8±58.1 |
| HDL (mg/dL) | 44.9±10.6 | 45.9±15.0 |  | 44.2±10.1 | 43.8±11.3 |
| LDL (mg/dL) | 164.9±53.7 | 126.5±32.3 |  | 148.1±61.4 | 149.0±49.2 |
| Insulin (IU/mL) | 14.1±4.6 | 11.5±3.6 |  | 12.8±5.1 | 13.4±7.3 |
|  |  |  |  |  |  |

Study from a randomized crossover design, the subjects were randomly received vitamin C or placebo for six weeks, followed by wash out period for six week and then received the other one for six week. Blood samples were collected at pre-and post-supplementation for each phase. Data represented as mean ± S.D. P-value was calculated using one way ANOVA and paired t-test. *= Significant difference from post-vitamin C supplementation compared to pre-vitamin C supplementation. ^#^= Significant difference from post-placebo supplementation compared to pre-placebo supplementation.

Abbreviations: BMI, body mass index; F_2_IsoPs, F_2_-Isoprostanes; FBS, fasting blood sugar; HbA1c, glycated haemoglobin; HDL, high-density lipoproteins; LDL, low- density lipoproteins MDA, malondialdehyde.
