# Supplementary material for: Vitamin C supplementation reduces expression of circulating miR-451a in subjects with poorly controlled type 2 diabetes mellitus and high oxidative stress
Source: PeerJ. 2021 Feb 4;9:e10776. doi: 10.7717/peerj.10776 (PMC7868066; doi:10.7717/peerj.10776)
Supplement: Supplemental Information 7 — Details of the project followed the Miame checklist. [file peerj-09-10776-s007.docx]

**Miame Checklist**

**Part 1 Experiment description**

 - Blood samples were collected from poorly controlled type 2 diabetic patients at before and after receiving 1,000 mg/day vitamin C for six weeks.

- Changed miRNA expression at post- supplementation were analyzed compared to pre- supplementation in 5 participants.

- The significantly expressed miRNAs from NanoString platform were next validated by qRT-PCR.

**Part 2 Array design**

 We used the Nanostring human miRNA panel which is a customed-commercial platform to detect miRNA expressions.

-         [nCounter® Human v3 miRNA Expression Assay](https://www.nanostring.com/products/mirna-assays/mirna-panels) from NanoString Technologies which is digitally direct counts of each miRNA without the use of reverse transcription or amplification.

-         800 human miRNAs can be detected.

-         miRNA in the sample can be bound by specific oligonucleotide tags (miRtags). The direct molecular barcoding and digital detection of target molecules using a color-coded probe pair.

- The probe pair consists of a Reporter Probe, which carries the signal on its 5’ end, and a Capture Probe, which carries a biotin on its 3’ end.

- The complexity of the color codes, comprised of four colors in six positions, allows a large diversity of targets present in the same sample to be individually resolved and identified during data collection.

-         The purified target/probe complexes were eluted off the beads and immobilized on the nCounter SPRINT Cartridge for data collection.

- 6 positive controls, 8 negative controls, 3 ligation positive controls, 3 ligation negative controls, and spike-in controls, cel-miR-39 and cel-miR-254, were used for quality control and normalization.

**Part 3 Samples**

 - Plasma sample from EDTA blood of poorly controlled type 2 diabetic patients who received vitamin C treatment for six weeks

- Total RNA extraction following the miRNeasy Serum/Plasma kit’s protocol (Qiagen, Germany) from 200 ul of the plasma sample

- Target miRNAs in sample were ligated with specific oligonucleotide tags (miRtags)

- miRNA sample prep protocol including annealing step, ligation step, purification step and hybridization step.

- Sample preparation for the detection is shown in Figure 1.

**Part 4 Hybridizations**

- Hybridization step was followed the manufacturer’s protocol as summarized in Figure 2.

- Each sample was separated into an individual tube but used reagents from the same master mix prep.

- Hybridization time was 12 hours.

- After hybridization, excess probes were washed away using a two-step magnetic bead-based purification that performed by The SPRINT Profiler instrument.

**Part 5 Measurements**

 - The nCounter SPRINT Profiler instrument was used for both post hybridization processing and data collection (Instrument name: 150p0007, Software version: 2.2.1.10).

- This nCounter Digital Analyzer is a multi-channel epifluorescence scanner specifically configured for use with NanoString’s nCounter Cartridges.

- The Digital Analyzer is a Class 1 laser product. The instrument contains an internal Class 2 laser barcode reader.

- It collects data by taking images of the immobilized fluorescent reporters in the sample cartridge with a CCD camera through a microscope objective len.

- 196 fields of view (FOV) were collected per flow cell (cartridge lane).

- Images were processed and each lane produced one RCC (Reporter Code Count) file. The zipped RCC files were downloaded via USB flash drive and imported into the nSolver^TM^ v4.0 analysis software for QC and analysis.

- Number of miRNA expression from NanoString represents number of miRNA counts. Normalized ratios can be analyzed by nSolver analysis software.

- Run data were exported as a comma separated values (CSV) format file that can be opened by Microsoft excel.

**Part 6 Normalization controls**

 All of our samples were done in one cartridge at the same time. We aimed to compare miRNA expression changes between pre-and post-supplementation. All data had no any flags. Background thresholding by count value > 100 counts were set to eliminate all targets with low expression. Normalization included two steps as described below

1. A positive control normalization; 6 spiked in positive controls were firstly calculated to adjust variations across samples, lanes, cartridges, days, and user techniques and processes.

2. A CodeSet content normalization was next calculated using reference gene, spike-in controls miR-254, to adjust for differences in analyte abundance and/or analyte quality across samples.

The analyses were analyzed using the NanoString’s nSolver Analysis Software or other data analysis and visualization software packages.


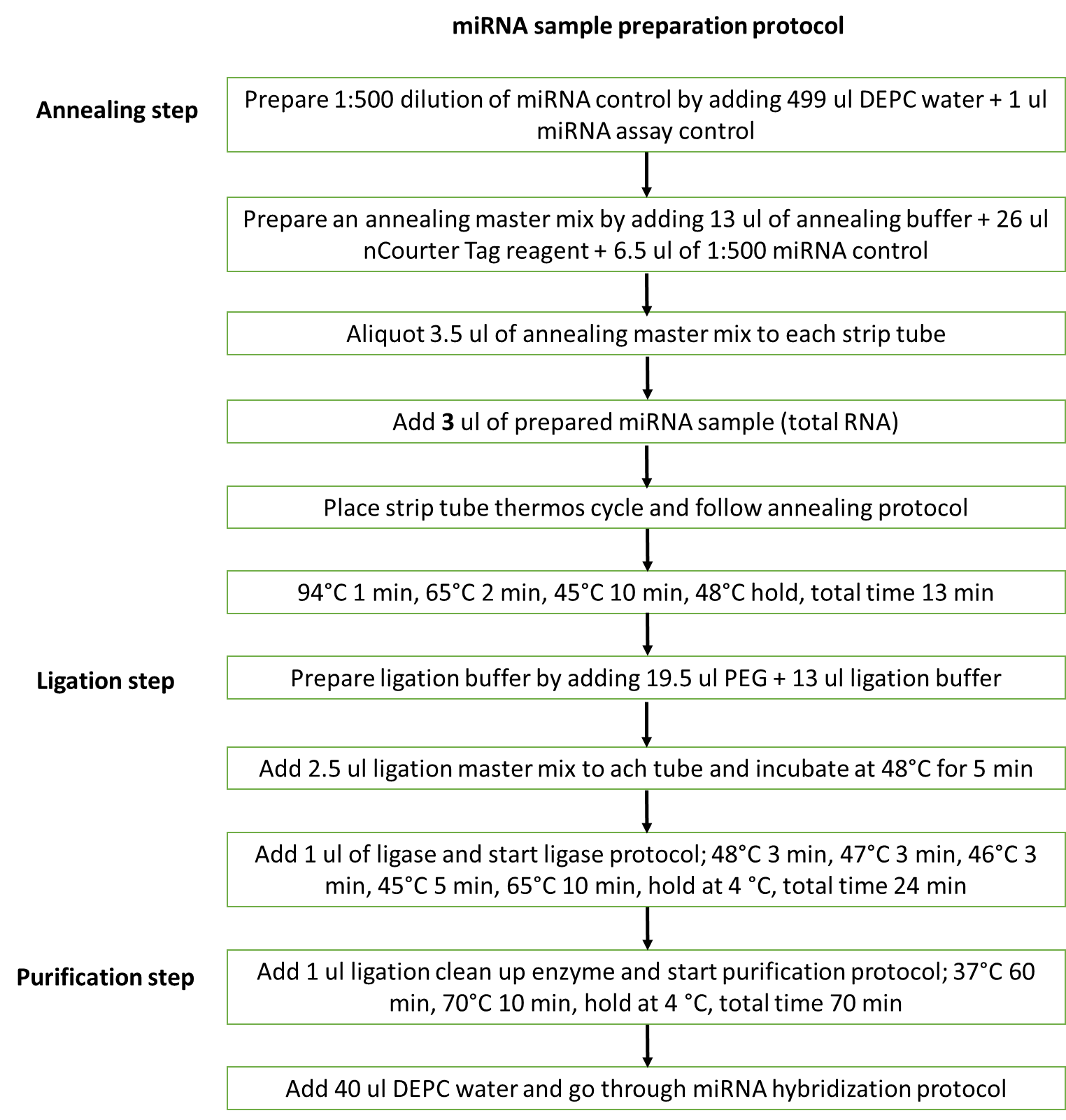


**Figure 1 miRNA sample preparation protocol.**


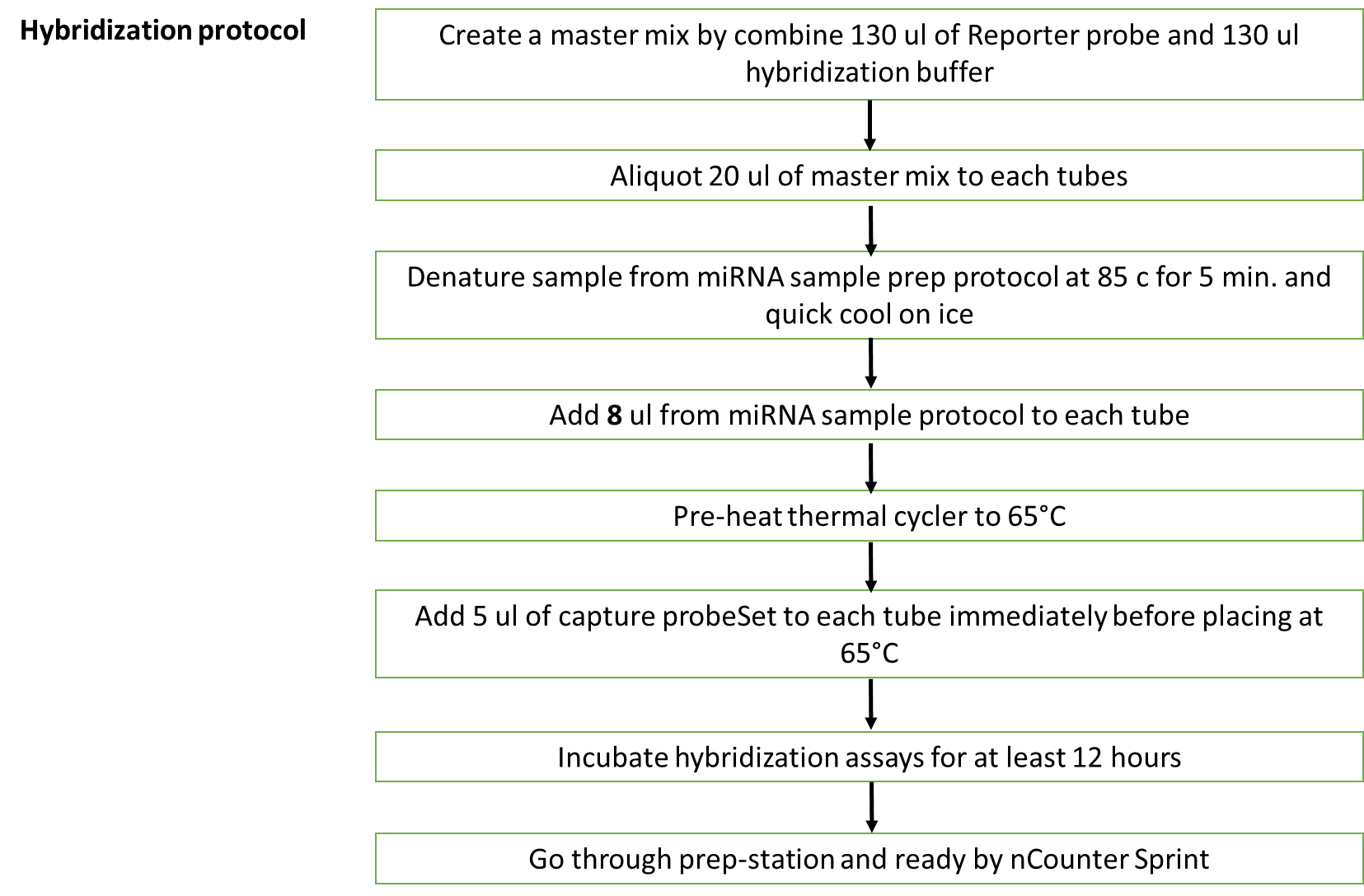


**Figure 2 Hybridization protocol.** Final volume of each tube was 33 ul. The reagents included 10 ul reporter code set + 10 ul hybridization buffer + 8 ul miRNA sample prep protocol + 5 ul capture probe set.
